# Monkeypox Virus Antibodies in Healthy Persons after Vaccination with MVA-BN, United Kingdom 

**DOI:** 10.3201/eid3202.251553

**Published:** 2026-02

**Authors:** Victoria H. Sheridan, Craig W. Duffy, Jake Dunning, Lance Turtle, Julian A. Hiscox, Krishanthi S. Subramaniam, on behalf of the ISARIC4C Investigators

**Affiliations:** University of Liverpool, Liverpool, UK (V.H. Sheridan, C.W. Duffy, L. Turtle, J.A. Hiscox, K.S. Subramaniam); University of Oxford, Oxford, UK (J. Dunning); Royal Liverpool University Hospital, Liverpool (L. Turtle)

**Keywords:** mpox, monkeypox virus, viruses, neutralization, MVA-BN vaccination, zoonoses, United Kingdom

## Abstract

A 2-dose regimen of the vaccine modified vaccinia Ankara–Bavarian Nordic (MVA-BN) can generate neutralizing antibodies for monkeypox virus clades Ib and IIb. We observed higher response to clade IIb; that result provides evidence that MVA-BN vaccination can induce cross-neutralizing antibodies for monkeypox virus clade Ib as well as for clade IIb.

Mpox is a zoonotic viral disease caused by monkeypox virus (MPXV), which is divided into clades I and II; clade II is subdivided into subclades IIa and IIb (*1*,*2*). In 2023 a new subclade of clade I, termed clade Ib, emerged in the Democratic Republic of the Congo (DRC). Since the first human case identified in August 1970 in DRC, mpox has been reported in 11 countries in Africa; in 2022 a global outbreak occurred in nonendemic areas caused by the clade IIb strain (*1*). More recently, the emergence of clade Ib, designated a public health emergency of international concern in August 2024 and associated with increased disease severity and mortality rate, particularly among children, posed a substantial public health threat (*3*). The World Health Organization recommends that persons at high risk of contracting mpox, especially during an outbreak, be vaccinated (*2*) with the modified vaccinia Ankara-Bavarian Nordic (MVA-BN) smallpox vaccine, a live attenuated vaccine (*1*).

Evidence demonstrates that vaccination with MVA-BN can generate low levels of neutralizing antibodies for clade IIb and clade Ia (*4*,*5*). In the United Kingdom, 1 dose of MVA-BN gives short-term protection of 78% against mpox, predominantly in men who have sex with men (*6*). Whether vaccination can also induce neutralizing antibodies for clade Ib has not been addressed. We recruited a convenience sample of healthcare workers (n = 25) vaccinated with MVA-BN for occupational exposure to mpox to measure neutralizing antibodies for clades Ib and IIb using a plaque reduction neutralization test (PRNT). 

The importance of complement in relation to neutralization levels has been reported for MPXV (*7*) and other viruses (*8*). To assess the contribution of complement in our cohort, we exposed serum samples to different conditions: heat inactivation (HI), HI supplemented with guinea pig serum as a complement source, and non-HI. We found, as previously reported (*7*), that complement is required for neutralization of MPXV in vitro (Figure, panel A). We detected no significant difference in MPXV neutralization between HI serum in the presence of a complement source and non-HI (p = 0.0625 by Wilcoxon signed-rank test). On the basis of those data, we used non-HI serum for the remainder of the experiments.

**Figure:**
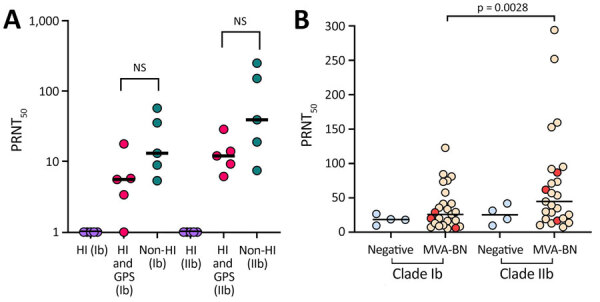
Titer results in study of monkeypox virus antibodies in healthy persons after MVA-BN vaccination, United Kingdom. PRNT_50_ titers for participants vaccinated with 2 doses of MVA-BN vaccine demonstrated neutralizing antibody responses to monkeypox virus clade Ib and clade IIb. A) Assessment of the contribution of complement on neutralization illustrating the different conditions tested: purple, HI; red, HI and GPS; blue, non-HI. Mann Whitney U test used to determine p values. B) PRNT_50_ values for clades Ib and IIb. Wilcoxon matched pairs signed rank test used to determine p values. Blue indicates negative controls; yellow, vaccine recipients; red, participants with underlying conditions. Each data point represents the geometric mean titer of 2 experimental replicates; horizontal black lines indicate medians. PRNT_50_ values were determined using Probit regression. GPS, guinea pig serum; HI, heat-inactivation; MVA-BN, modified vaccinia Ankara–Bavarian Nordic vaccine; NS, not significant; PRNT_50_, log of 50% endpoint plaque reduction neutralization test.

We measured neutralization of MPXV clade Ib and IIb in non-HI serum samples from 25 vaccine recipients. We included 4 persons who did not receive the MVA-BN vaccine but were immunized with another live attenuated vaccine, IMOJEV (Substipharm, https://www.substipharm.com), as controls. In the MVA-BN vaccine group, 3 participants had underlying conditions of multiple sclerosis, psoriasis, or asthma (Table). The median PRNT_50_, which is defined as the reciprocal of the serum dilution that results in 50% reduction in virus plaques, was 25.9 (interquartile range [IQR] 10.05–49.7) for clade Ib and 44.8 (IQR 19.55–89.4) for clade IIb. Comparisons across these samples demonstrated that 2 doses of MVA-BN generated greater neutralization of MPXV clade IIb than of clade Ib, a difference we found to be statistically significant (p = 0.0028 by Wilcoxon signed-rank test) (Figure, panel B). The difference in neutralizing antibody titers is small, and the relevance for clinical protection is uncertain. The protective threshold for MPXV neutralizing antibodies is not defined; case–control studies could define antibody-specific correlates of protection. The negative controls did exhibit low levels of nonspecific neutralization (PRNT_50_ 18.5 for clade Ib and 25 for clade IIb), which were lower than those observed in the MVA-BN vaccine group.

**Table:** Demographics of the participant cohort in study of monkeypox virus antibodies in healthy persons after MVA-BN vaccination, United Kingdom*

Characteristic	MVA-BN vaccine recipients, N = 25
Median age, y (IQR)	39 (30–45)
Sex, no. (%)	
M	9 (36)
F	16 (64)
Ethnicity, no. (%)	
White	20 (80)
Asian	3 (12)
Latin	2 (8)
Underlying conditions, no.	
Multiple sclerosis	1
Psoriasis	1
Asthma	1

*IQR, interquartile range; MVA-BN, modified vaccinia Ankara–Bavarian Nordic.

Our results showed low levels of MPXV neutralization from MVA-BN vaccination, consistent with previous studies (*4*,*5*,*9*). We found that neutralization of clade Ib was lower than for clade IIb. Although our study is limited by relatively small sample size, we demonstrated neutralization of MPXV clade Ib in vaccine recipients without a history of mpox and compare those results with clade IIb neutralization. Moreover, given that the study cohort included healthcare workers at highest risk for exposure, evidence of vaccine-associated neutralization is relevant to determine policies regarding future vaccine rollouts.

MPXV neutralization is known to require complement (*7*). We observed low levels of neutralization when guinea pig serum was added to virus and when pooled human plasma was added to virus (data not shown), highlighting the nonspecific effect that foreign complement sources can have on MPXV neutralization; guinea pig serum alone exhibits neutralization activity against mumps virus compared with purified antibodies alone (*10*). Therefore, our approach was to use non–heat-inactivated serum to measure MPXV neutralization as described previously (*9*).

The low levels of neutralization we observed, particularly against MPXV clade Ib, suggest that vaccination with MVA-BN can confer moderate protection against disease caused by that clade. The durability of those responses, and whether a third dose is required to enhance protection against mpox clade Ib infections (*4*,*5*,*9*), were beyond the scope of our study.

Appendix Additional information from study of monkeypox virus antibodies in healthy persons after vaccination, United Kingdom.
