# Supplementary material for: Monkeypox Virus Antibodies in Healthy Persons after Vaccination with MVA-BN, United Kingdom
Source: Emerg Infect Dis. 2026 Feb;32(2):302–5. doi: 10.3201/eid3202.251553 (PMC12928222; doi:10.3201/eid3202.251553)
Supplement: Appendix — Additional information from study of monkeypox virus antibodies in healthy persons after vaccination, United Kingdom. [file 25-1553-Techapp-s1.pdf]

Article DOI: https://doi.org/10.3201/eid3202.251553

Monkeypox Virus Antibodies in Healthy Persons after Vaccination with MVA-BN, United Kingdom

Appendix

Methods

Study Design and Participants

Blood samples were obtained from twenty-five healthcare workers with occupational exposure to mpox who received 2 doses of the Modified Vaccinia Ankara (MVA) Bavarian Nordic (MVA-BN) vaccine via subcutaneous or intradermal routes (Table). Six of the twenty-five study participants were sampled at more than 1 month post dose 2 (median = 13.5 months, IQR = 10 – 38). The remaining subjects all provided a serum sample at 28 days post MVA-BN dose 2. Four individuals who did not receive MVA-BN but were immunised with IMOJEV®, a live attenuated vaccine against Japanese Encephalitis Virus (JEV) were used as negative controls to assess nonspecific neutralisation. None of the study subjects had received a previous smallpox vaccination. Vaccine recipients were recruited into the Acute Virus Immunity Study (AVIS; REC 16/NW/0170) at the University of Liverpool. All participants provided written informed consent before sample collection.

Viruses and Cells

The Clade IIb mpox virus, 2022, Slovenia ex Gran Canaria was obtained from the European Virus Archive Global. MPXV Clade Ib, 2024, London ex East Africa (October 2024) was isolated from a clinical sample collected under the ISARIC Clinical Characterization Protocol-UK (approved in England by Oxford Research Ethics Committee, ref 13/SC/0149). We passaged virus stocks in Vero E6 cells (African green monkey kidney, C1008 Public Health England PHE – UK Health Security Agency (UKHSA)) maintained in Dulbecco’s Modified Eagle Medium (DMEM) with 2% heat-inactivated fetal bovine serum and 0.05mg/ml gentamicin and harvested 72 h post inoculation.

Virus Sequencing

DNA was extracted from MPXV stocks using Zymo DNA/RNA viral extraction kit. The NextGen PCR MPXV Sequencing Library Prep Kit (MBS) was used in conjunction with Oxford Nanopore Rapid Sequencing DNA kit V14 - barcoding (SQK-RBK114.24) to amplify viral DNA (*1*). Mpox PCR amplicons were sequenced on an Oxford Nanopore P2 R10.4 flow cell following manufacturer's instructions. Low quality reads (average q-score <9) were discarded during base calling. Reads passing initial QC were trimmed with porechop_abi to remove barcodes. Reads were mapped to MPOX clade 1 and clade 2 reference genomes using minimap2 v2.26 with map-ont presets (*2*). Consensus sequences were created from individual bam files using SAMtools v1.18 consensus using the Bayesian model (*3*). Sequences were mapped to both clades to confirm that the choice of reference sequence did not affect clade assignment during construction of the tree.

Consensus sequences were combined with MPOX Clade I and II reference sequences NC003310 and NC063383 and 100 previously published MPOX sequences with known clade assignments. Previously published GenBank sequences and clade assignments were retrieved from the Nextstrain MPOX instance (https://github.com/nextstrain/mpox) and filtered to retain sequences with >95% coverage relative to the reference (*4*). 25 sequences were randomly selected for each of the major subclades (Ia, Ib, IIa, IIb) for inclusion in the tree. Sequences were aligned with MAFFT v7.525 using automatic parameter determination (*5*). An approximate maximum-likelihood neighbor joining tree was constructed with FastTree v2.1.11 under a Jukes-Cantor GTR model of nucleotide evolution, with the tree visualized in R using ggtree (*6*,*7*) (Appendix Figure).

Plaque Reduction Neutralisation Titers (PRNT)

PRNTs were performed using Vero E6 cells. Sera were diluted 1:4 in DMEM (2% FBS; 0.05 mg/mL gentamicin) followed by serial 2-fold dilutions. MPXV at 700 PFU/mL was added to an equal volume of diluted sera and incubated at 37°C for 1 h. In some experiments, 10% guinea pig serum (a source of complement), was added to the starting serum dilution (final concentration 5% when mixed with MPXV). The virus-serum dilution was inoculated onto Vero E6 cells in duplicate. Cells were incubated for 72 h at 37°C and 5% $CO2$ before being fixed with 10% formalin and stained with crystal violet solution. The 50% plaque reduction titer ($PRNT_{50}$) was determined via probit regression analysis using a script adapted from Bewley et al. 2021 (*8*).

In brief plaque counts were entered into Microsoft Excel, under four column headers, labeled as conc, success, failure and VOC (Virus Only Control). In the 'conc' column, the reciprocal of the dilutions used for the sample being analyzed was added (4, 8, 16, 32 etc.). In the 'failure' column, the average plaque counts from two wells containing virus and serum was input alongside the corresponding serum dilution. In the 'VOC' column, the average plaque count from the virus control was input. To calculate the 'success' rate, the plaque count (failure) was subtracted from the VOC. The worksheet was saved as a .csv file with the sample ID. The software package 'R' was used with the script available from Bewley et al. 2021. A graph was generated within R which calculated the median plaque reduction neutralising dose ($PRNT_{50}$) with confidence intervals. When a 50% reduction in number of plaques was not seen at 1:4 dilution a value of 0 was assigned to that sample.

Statistical Analysis

Statistical analysis was done using GraphPad Prism version 10 (GraphPad Software). The non-parametric Mann-Whitney U-test and Wilcoxon matched-pairs signed-rank tests were used to compare differences across the samples. P values less than 0.05 were considered significant.

References

1. Welkers M, Jonges M, van den Ouden A. Monkeypox virus whole genome sequencing using combination of NextGenPCR and Oxford Nanopore V.1. 2022 June [cited 2025 Jul 27]. ttps://doi.org/10.17504/protocols.io.n2bvj6155lk5/v1

2. Li H. Minimap2: pairwise alignment for nucleotide sequences. Bioinformatics. 2018;34:3094–100. PubMed https://doi.org/10.1093/bioinformatics/bty191

3. Danecek P, Bonfield JK, Liddle J, Marshall J, Ohan V, Pollard MO, et al. Twelve years of SAMtools and BCFtools. Gigascience. 2021;10:giab008. PubMed https://doi.org/10.1093/gigascience/giab008

4. Hadfield J, Megill C, Bell SM, Huddleston J, Potter B, Callender C, et al. Nextstrain: real-time tracking of pathogen evolution. Bioinformatics. 2018;34:4121–3. PubMed https://doi.org/10.1093/bioinformatics/bty407

5. Katoh K, Standley DM. MAFFT multiple sequence alignment software version 7: improvements in performance and usability. Mol Biol Evol. 2013;30:772–80. PubMed https://doi.org/10.1093/molbev/mst010

6. Price MN, Dehal PS, Arkin AP. FastTree 2—approximately maximum-likelihood trees for large alignments. PLoS One. 2010;5:e9490. PubMed https://doi.org/10.1371/journal.pone.0009490

7. Yu G, Smith DK, Zhu H, Guan Y, Lam TTY. ggtree: an R package for visualization and annotation of phylogenetic trees with their covariates and other associated data. Methods Ecol Evol. 2017;8:28–36. https://doi.org/10.1111/2041-210X.12628

8. Bewley KR, Coombes NS, Gagnon L, McInroy L, Baker N, Shaik I, et al. Quantification of SARS-CoV-2 neutralizing antibody by wild-type plaque reduction neutralization, microneutralization and pseudotyped virus neutralization assays. Nat Protoc. 2021;16:3114–40. PubMed https://doi.org/10.1038/s41596-021-00536-y

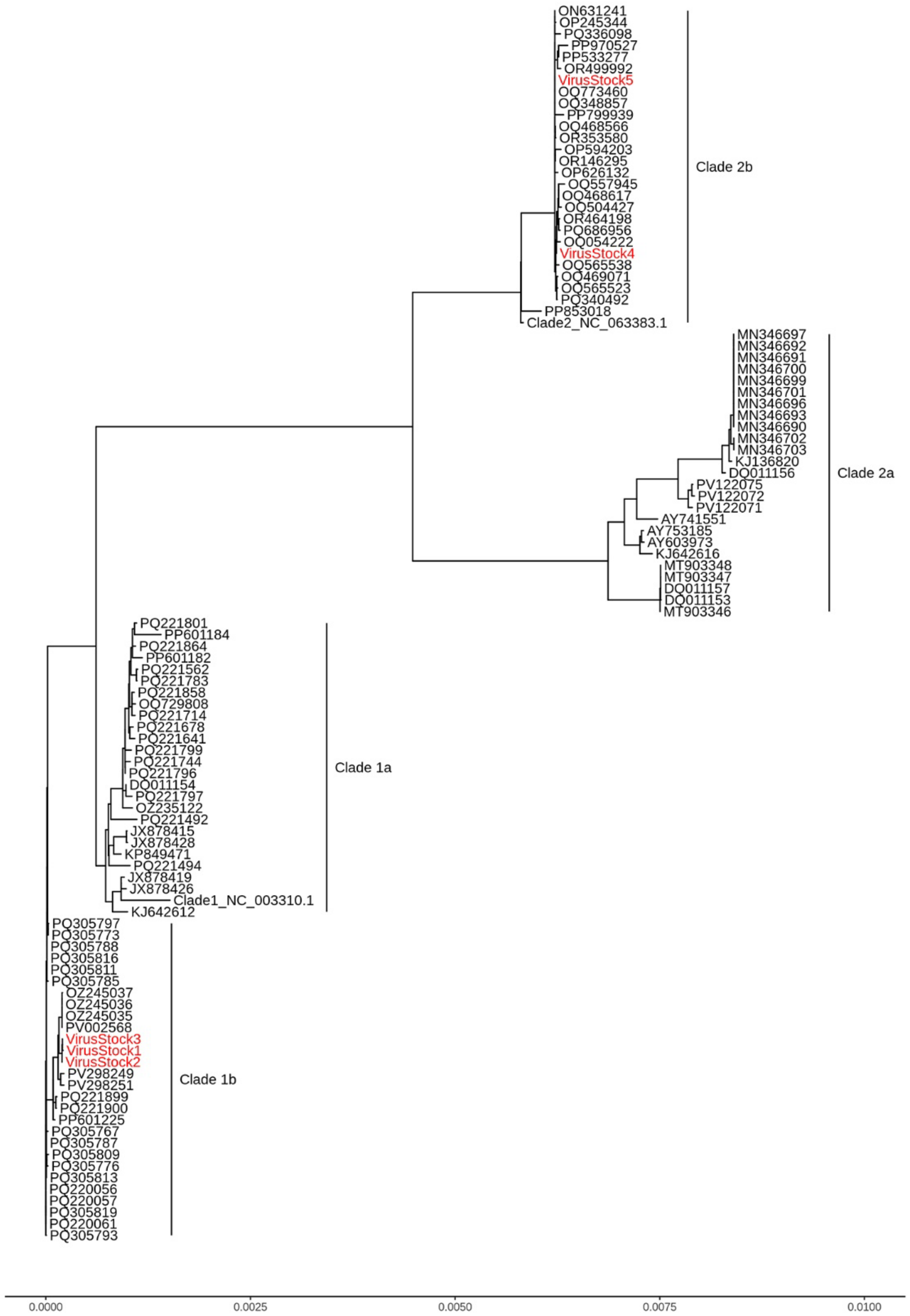

Appendix Figure. Phylogenetic tree of monkeypox virus strains used in study of monkeypox virus antibodies in healthy persons after vaccination, United Kingdom. Three Clade Ib and two Clade IIb virus stocks were sequenced using ONT. Sequences were aligned and plotted on a phylogenetic tree against known sequences of both mpox clades and subclades.
